# Using Machine Learning to Predict the Prognosis in Endometrial Cancer Patients Undergoing Fertility‐Sparing Treatment

**DOI:** 10.1002/cam4.71355

**Published:** 2025-11-20

**Authors:** Wenhan Yuan, Haidan Yin, Jianhong Liu, Ying Zheng

**Affiliations:** ^1^ Department of Gynecology and Obstetrics, West China Second University Hospital Sichuan University Chengdu Sichuan China; ^2^ Key Laboratory of Obstetrics and Gynecologic and Pediatric Diseases and Birth Defects of Ministry of Education, West China Second Hospital Sichuan University Chengdu Sichuan China; ^3^ School of Remote Sensing and Information Engineering Wuhan University Wuhan Hubei China

**Keywords:** endometrial cancer, fertility‐sparing treatment, machine learning, magnetic resonance imaging, prognostic model, radiomics

## Abstract

**Background:**

Endometrial cancer is a significant gynecological malignancy with rising incidence among women of reproductive age, necessitating effective fertility‐sparing treatments. Machine learning offers potential in enhancing prognostic assessments through radiomics.

**Objective:**

To develop and validate a machine learning‐based model integrating radiomics and clinical features to predict the prognosis of fertility‐sparing treatments in endometrial cancer patients.

**Methods:**

This retrospective study included 102 endometrial cancer patients who received fertility‐sparing treatment at West China Second University Hospital from November 2017 to December 2023. Patients were randomly divided into training (*n* = 81) and testing (*n* = 21) cohorts. The primary outcome was 6 month treatment response evaluated by endometrial sampling. MRI‐based radiomic features and clinical data were analyzed using logistic regression and other machine learning algorithms. A combined model was constructed by incorporating radiomics and clinical features. Model performances were assessed by the area under the receiver operating characteristic curve (AUC) analysis, and decision curve analysis (DCA) was used to estimate the models' clinical values.

**Results:**

Among 102 patients, 60 (58.8%) achieved complete remission (CR), whereas 42 (41.2%) did not (non‐CR). In total, 1197 radiomic features were extracted from MRI images. Thirteen radiomic features were deemed valuable by dimensionality reduction and selection. Among radiomic models, the logistic regression model was the most effective, showing high stability and accuracy, with AUCs of 0.875 in the training cohort and 0.852 in the test cohort. Ultimate clinical features selected by univariate and multivariable logistic regression constructed a clinical model. The combined model demonstrated superior performance with an AUC of 0.941 in the training cohort and 0.907 in the testing cohort. Combined model DCA revealed optimal clinical efficacy.

**Conclusion:**

The integrated model effectively predicts fertility preservation outcomes, offering a reliable noninvasive tool for clinicians. This approach may enhance personalized treatment strategies for young endometrial cancer patients.

## 1 Introduction

Endometrial cancer (EC) is a prevalent gynecological malignancy, ranking sixth among all cancers globally [1]. In recent years, the incidence of EC in women of reproductive age has steadily increased, with approximately 7% of cases occurring in women under the age of 45 [2, 3].

In recent years, an increasing number of studies have validated the safety and efficacy of high‐dose progestin conservative treatment for patients with early‐stage endometrioid endometrial carcinoma [4, 5, 6, 7, 8, 9, 10]. The primary objective of conservative treatment is to achieve complete remission (CR); however, the factors influencing response rates remain inadequately defined. These factors include the molecular characteristics of the disease, patient weight (with improved response rates observed in those with a body mass index (BMI) < 25 kg/m2), histological grade, and polycystic ovary syndrome (PCOS), among others, though evidence supporting their clinical utility remains limited [11, 12, 13, 14, 15]. While guidelines recommend that the absence of myometrial invasion be confirmed by magnetic resonance imaging (MRI) before opting for a fertility‐sparing approach [16], retrospective case series suggest that patients with endometrial cancer and myometrial invasion may also be candidates for fertility‐sparing treatment [13, 17]. Thus, accurately identifying patients suitable for fertility‐sparing treatment is of paramount importance.

In recent years, machine learning technology has advanced rapidly, particularly with significant breakthroughs in image processing and analysis, demonstrating considerable advantages in disease prediction and prognosis. It has shown substantial potential in detecting brain hemorrhages on cranial CT scans [18], diagnosing skin cancer [19], and identifying diabetic retinopathy [20]. Radiomics, a subfield of machine learning, is an emerging and promising discipline that quantitatively characterizes tumors by extracting and analyzing numerous image features using high‐throughput techniques [21]. In the past few years, a growing body of research has explored the application of radiomics in endometrial cancer [22, 23, 24]. These studies suggest that machine learning can assist in addressing clinical challenges and optimizing treatment for endometrial cancer patients. However, to our knowledge, no studies have yet examined the application of different machine learning models in predicting the prognosis of fertility‐sparing treatments for endometrial cancer patients.

In this study, we employed various machine learning approaches to develop and validate an MRI‐based combined model integrating radiomics and clinical features to predict the prognosis (remission status) of endometrial cancer patients undergoing fertility‐sparing treatment after 6 months.

## 2 Materials and Methods

### 2.1 Study Design and Study Population

This retrospective study included patients who received fertility‐sparing treatment at West China Second University Hospital between November 2017 and December 2023. The study was reviewed and approved by the institutional review board of the West China Second Hospital of Sichuan University (IRB No. 2023[024]). Written informed consent was obtained from all the participants prior to the enrollment of this study. Medical records were utilized to collect data on each patient's clinical characteristics and oncological outcomes.

The inclusion criteria were as follows: (1) age ≤ 45 years; (2) a strong desire for fertility preservation; (3) histological diagnosis of endometrioid adenocarcinoma (grade 1 or grade 2) confirmed by designated gynecological pathologists; (4) positive progesterone receptor status in immunohistochemistry; (5) underwent enhanced abdomen‐pelvic MRI; (6) patient response evaluated through endometrial sampling via hysteroscopy 6 months posttreatment.

The exclusion criteria included: (1) the presence of malignancies at other sites or Lynch syndrome; (2) incomplete clinical data; (3) MRI quality that did not meet the analysis requirements.

All patients received fertility‐sparing therapy based on progestin regimens. The treatment options included: (1) oral progestin therapy with medroxyprogesterone acetate (MPA, 250–500 mg/day) or megestrol acetate (MA, 160–480 mg/day); (2) levonorgestrel‐releasing intrauterine system (LNG‐IUS, Mirena 52 mg); (3) gonadotropin‐releasing hormone agonist (GnRH‐a, 3.75 mg every 4 weeks) combined with the above regimens. Treatment continued for 6 months, and endometrial biopsy via hysteroscopy was performed to evaluate response.

**FIGURE 1:**
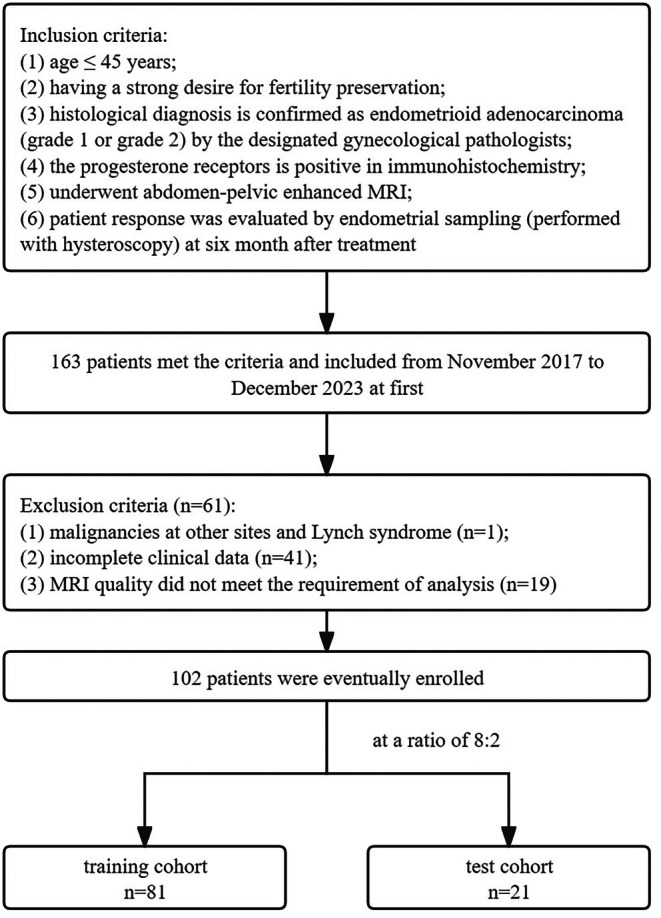
Flowchart for selecting the study population.

Patients were randomly assigned to a training cohort and a test cohort in an 8:2 ratio (see Figure 1 for details).

### 2.2 Clinical Data Analysis

The patients' imaging and clinical data were obtained from routine clinical records and the Picture Archiving and Communication System (PACS) at our hospital. We conducted a retrospective analysis of various clinical parameters, including age, body mass index (BMI), parity, and comorbidities such as polycystic ovary syndrome (PCOS), thyroid disease, diabetes, hypertension, and insulin resistance (IR), as well as menstrual status. Laboratory data were collected and analyzed for hemoglobin (Hb), platelet count (PLT), neutrophil count (NEUT), lymphocyte count (LYMPH), monocyte count (MONO), tumor markers (including carbohydrate antigen 125 (CA125), carbohydrate antigen 199 (CA199), alpha‐fetoprotein (AFP), and HE4), and liver and kidney function indicators, including albumin (ALB), alanine aminotransferase (ALT), aspartate aminotransferase (AST), total bilirubin (TB), blood urea (URE), and serum creatinine (CRE). Pathological immunohistochemical indicators included p53 status, Ki‐67, myometrial invasion, and histological grade; imaging characteristics included the maximum tumor diameter. The primary outcome is the patient's response, assessed through endometrial sampling performed via hysteroscopy, 6 months after treatment.

### 2.3 Image Acquisition and Image Segmentation

All MR examinations were conducted using 1.5 T and 3.0 T scanners (GE Signa HDXt, Siemens Prisma, and Siemens Aera) equipped with eight‐channel phased‐array abdominal coils. Each patient underwent MR scanning according to a standardized protocol. In this study, uterus‐axial T2‐weighted images (T2WI) were acquired for image segmentation.

**FIGURE 2:**
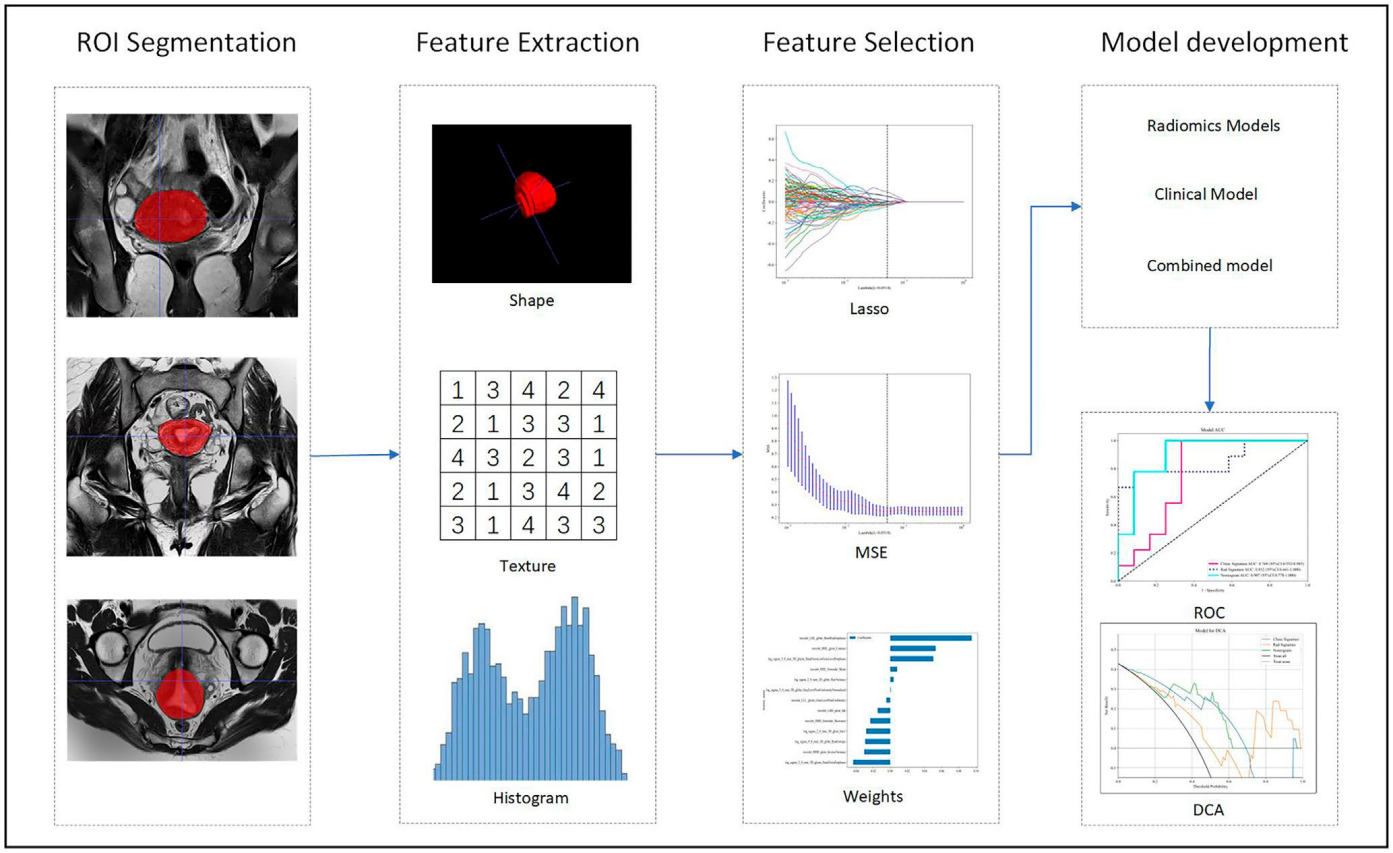
Workflow of the study.

All images were saved using standard soft tissue settings (window width, 1200 HU; window level, 600 HU) and stored in Digital Imaging and Communications in Medicine (DICOM) format. Two radiologists, with 10 and 20 years of diagnostic experience, respectively, manually segmented the regions of interest (ROIs) using ITK‐SNAP software (version 3.8.0, http://www.itksnap.org/). On axial uterine T2WI, ROIs were manually contoured layer by layer to encompass the primary tumor, intratumoral components (hemorrhage, necrosis, cystic change), and immediately adjacent invaded myometrium. A complete schematic is provided in Figure 2.

Images and data were preprocessed through resampling and standardization to ensure the repeatability of the results. Intra‐ and interobserver reproducibility was assessed using the intraclass correlation coefficient (ICC). Thirty‐five patients (21 complete remission and 14 noncomplete remission) were randomly selected, and radiologist 1 re‐segmented the ROIs after 2 months. An ICC greater than 0.8 indicated good consistency.

### 2.4 Feature Extraction and Selection

Before feature extraction, to mitigate imaging variability between different MRI scanners, images are normalized to ensure that all gray‐level values fall within the range of 0 to 600. The normalized MR images are subsequently processed using various built‐in filters, including gradient, exponential, logarithmic, square, square root, wavelet, and Laplacian of Gaussian (LOG) filters, to generate derived images.

Radiomic features were extracted from radiologist‐defined ROIs using the open‐source Python package Pyradiomics (https://pypi.org/project/pyradiomics/). The extracted features were categorized into three groups: (1) geometry, (2) intensity, and (3) texture. Geometry features describe the three‐dimensional shape characteristics of the tumor. Intensity features represent the first‐order statistical distribution of voxel intensities within the tumor. Texture features capture patterns or the second‐ and higher‐order spatial distributions of intensities. Texture features were extracted using several methods, including gray‐level co‐occurrence matrix (GLCM), gray‐level dependence matrix (GLDM), gray‐level run length matrix (GLRLM), gray‐level size zone matrix (GLSZM), and neighborhood gray‐tone difference matrix (NGTDM) [25]. All features were standardized using Z‐scores.

**FIGURE 3:**
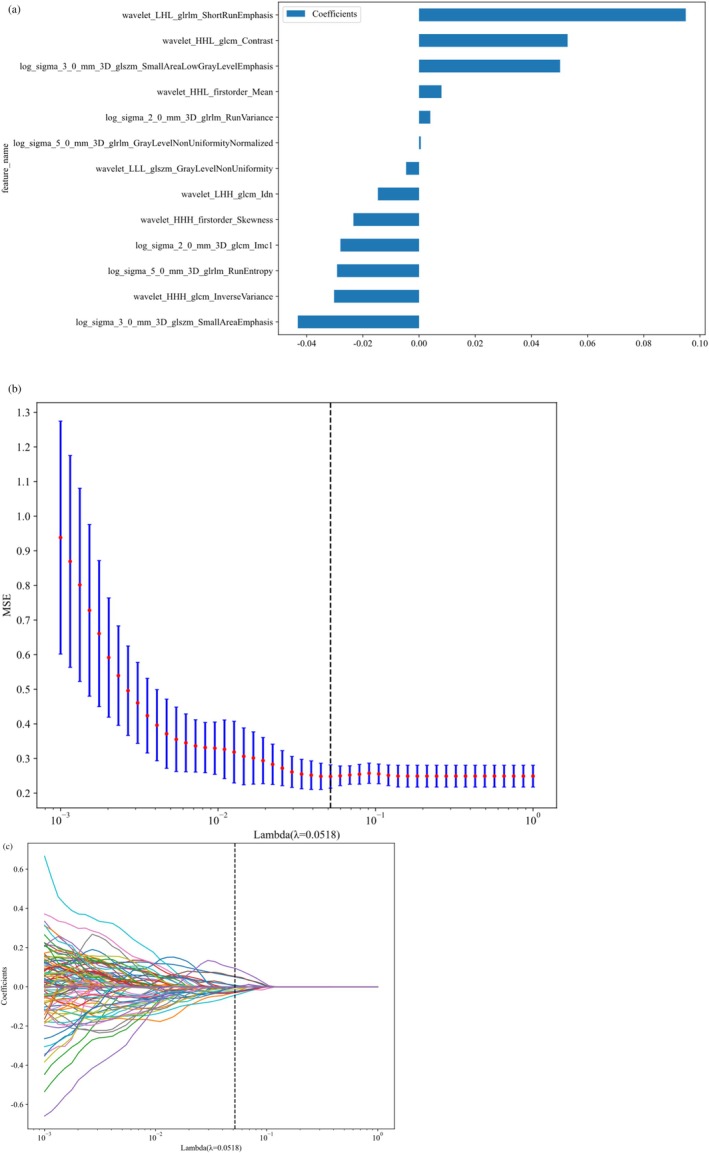
Radiomic feature selection based on LASSO regression model. (a, b) Ten‐fold cross‐validated coefficients and 10‐fold cross‐validated MSE. (c) Bar chart of feature weight for the LASSO regression model.

Dimensionality reduction and feature selection methods for the radiomic features in the training cohort were conducted as follows: First, the 1197 extracted features underwent analysis of variance (ANOVA), with features exhibiting an ICC score greater than 0.8 and statistical significance being selected (628 features remain). Second, for features with high reproducibility, the Spearman rank correlation coefficient was used to assess the correlation between features, retaining only one of any two features with a correlation coefficient greater than 0.9 (176 features remain). Next, the least absolute shrinkage and selection operator (LASSO) regression model was employed to select the most representative features, followed by 10‐fold cross‐validation [26] (13 features remain) (Figure 3). All feature selection processes were performed on the training cohort and subsequently applied to the testing cohort.

### 2.5 Development of Radiomics Signature, Clinical and Combined Models

To construct the clinical model, univariate logistic regression analysis was initially performed on each clinical predictor variable. Statistically significant features were then subjected to multivariate logistic regression analysis to identify the final predictor variables for model development. The odds ratios (ORs) and 95% confidence intervals (CIs) for each factor were calculated.

In our study, six widely used machine learning algorithms were employed to develop the radiomics model, including logistic regression (LR), support vector machine (SVM), K‐nearest neighbors (KNN), random forest (RF), extreme gradient boosting (XGBoost), and light gradient boosting machine (LightGBM). The predictive performance of these models was evaluated using the area under the receiver operating characteristic (ROC) curve (AUC), accuracy, sensitivity, specificity, positive predictive value (PPV), and negative predictive value (NPV). The best‐performing radiomics models were then selected to construct a combined model incorporating both radiomics and clinical features [27].

The model's performance was assessed and validated using both the training and testing cohorts, focusing on metrics such as AUC, accuracy, sensitivity, specificity, PPV, and NPV. Decision curve analysis (DCA) was used to quantify the net benefits across different threshold probabilities in both cohorts, thereby evaluating the model's clinical utility.

### 2.6 Statistical Analysis

Statistical analyses were conducted using SPSS software (version 26.0, IBM), R software (version 4.1.2; https://www.r‐project.org), and Python software (version 3.9; http://www.python.org). The Shapiro–Wilk test was used to evaluate whether the continuous variables were normally distributed. A Student's t‐test was used to analyze continuous variables conforming to a normal distribution. The Mann–Whitney U test was used to analyze the continuous variables with non‐normal distributions. Continuous variables were expressed as mean ± standard deviation (x ± s). Categorical variables were analyzed using the chi‐square test or Fisher's exact test and expressed as ratios. The forward stepwise selection method was applied in multivariable logistic regression analysis using the Akaike information criterion (AIC) as the selection criterion. A two‐tailed *p* < 0.05 was considered statistically significant.

## 3 Results

### 3.1 Patients' Population and Clinical Data

A total of 102 patients were enrolled in our study, with 81 in the training cohort and 21 in the testing cohort. The results revealed no significant differences between the training and testing groups, with the clinical characteristics of the patients summarized in Table 1. The primary outcome was 6 month complete remission (CR) vs. non‐CR on hysteroscopic biopsy. In total, CR occurred in 60/102 (58.8%) patients (training: 51/81, 63.0%; testing: 9/21, 42.9%). Among the training cohort, significant differences were identified between CR and non‐CR patients in HE4 levels, maximum tumor diameter, total bilirubin, PCOS, insulin resistance, Ki‐67 levels, and myometrial invasion. In the testing cohort, significant differences were found in PCOS, maximum tumor diameter, AFP, ALB, and HE4 levels. Univariate and multivariate logistic regression analyses demonstrated that HE4 levels, myometrial invasion, PCOS, insulin resistance, and maximum tumor diameter were independent predictors of successful fertility preservation (Table 2). A clinical model was developed based on these five factors. Additionally, patients who did not achieve complete remission after 6 months of treatment were more likely to present with PCOS (OR, 0.469; 95% CI, 0.286–0.770), insulin resistance (OR, 0.540; 95% CI, 0.329–0.886), myometrial invasion (OR, 0.506; 95% CI, 0.299–0.855), larger tumor diameters (OR, 0.194; 95% CI, 0.062–0.608), and elevated HE4 levels (OR, 0.431; 95% CI, 0.232–0.799).

**TABLE 1:** Clinical characteristics in the training and test cohorts.

Variable		Training cohort (*n* = 81)			Testing cohort (*n* = 21)		
		Non‐CR (*n* = 30)	CR (*n* = 51)	*p*	Non‐CR (*n* = 12)	CR (*n* = 9)	*p*
Age (years)		29.83 ± 4.43	30.59 ± 4.55	0.468	29.17 ± 4.61	30.44 ± 3.84	0.508
BMI (kg/m^2^)		26.35 ± 6.24	25.03 ± 5.16	0.457	23.96 ± 5.31	28.09 ± 4.43	0.074
HB (g/L)		119.17 ± 14.87	121.31 ± 21.64	0.357	124.58 ± 26.88	137.44 ± 10.11	0.433
PLT (10^9^/L)		283.30 ± 81.70	281.18 ± 85.38	0.912	288.42 ± 66.01	279.33 ± 47.64	0.730
NEUT (10^9^/L)		5.11 ± 2.41	4.21 ± 1.44	0.160	3.88 ± 0.65	5.35 ± 2.17	0.069
LYMPH (10^9^/L)		1.94 ± 0.65	2.02 ± 0.81	0.925	2.00 ± 0.58	2.56 ± 0.81	0.109
MONO (10^9^/L)		0.45 ± 0.17	0.37 ± 0.14	0.072	0.39 ± 0.13	0.47 ± 0.11	0.153
CA125 (U/ml)		15.99 ± 11.62	13.78 ± 11.96	0.163	12.57 ± 5.13	15.48 ± 11.97	0.943
CA199 (U/ml)		16.24 ± 10.74	17.18 ± 13.20	0.814	13.75 ± 6.87	14.83 ± 10.60	0.779
AFP (ng/ml)		2.29 ± 1.29	2.82 ± 1.92	0.313	2.95 ± 1.57	1.96 ± 1.17	0.032*
HE4 (pmol/L)		43.63 ± 14.17	30.92 ± 9.68	< 0.001*	42.41 ± 13.77	28.06 ± 7.28	0.017*
ALB (g/L)		46.59 ± 5.37	46.86 ± 3.00	0.454	48.75 ± 1.90	46.60 ± 2.24	0.027*
ALT (U/L)		31.43 ± 21.35	28.43 ± 21.75	0.389	32.00 ± 23.31	34.33 ± 33.61	0.914
AST (U/L)		25.80 ± 11.77	25.45 ± 11.64	0.720	26.17 ± 10.65	28.78 ± 20.31	1
TB (umol/L)		10.68 ± 3.92	12.95 ± 4.72	0.015*	13.89 ± 4.26	10.46 ± 3.99	0.075
URE (mmol/L)		4.17 ± 1.36	4.35 ± 0.96	0.496	4.19 ± 1.14	5.00 ± 1.46	0.168
CRE (umol/L)		53.07 ± 8.82	55.04 ± 8.29	0.315	52.67 ± 9.99	52.33 ± 6.87	0.932
Max‐diameter (cm)		2.55 ± 1.58	1.14 ± 0.82	< 0.001*	2.52 ± 1.37	0.96 ± 0.59	0.007*
Parity	0	28 (93.33%)	48 (94.12%)	1	12 (100.00)	9 (100.00)	1
	≥ 1	2 (6.67%)	3 (5.88%)		Null	Null	
PCOS	With	11 (36.67%)	5 (9.80%)	0.008*	6 (50.00%)	Null	0.043*
	Without	19 (63.33%)	46 (90.20%)		6 (50.00%)	9 (100.00%)	
Thyroid disease	With	1 (3.33%)	1 (1.96%)	1	1 (8.33%)	Null	1
	Without	29 (96.67%)	50 (98.04%)		11 (91.67%)	9 (100.00%)	
Diabetes	With	4 (13.33%)	4 (7.84%)	0.678	1 (8.33%)	3 (33.33%)	0.377
	Without	26 (86.67%)	47 (92.16%)		11 (91.67%)	6 (66.67%)	
Hypertension	With	null	1 (1.96%)	1	1 (8.33%)	1 (11.11%)	1
	Without	30 (100.00%)	50 (98.04%)		11 (91.67%)	8 (88.89%)	
Insulin resistance	With	19 (63.33%)	12 (23.53%)	< 0.001*	7 (58.33%)	1 (11.11%)	0.079
	Without	11 (36.67%)	39 (76.47%)		5 (41.67%)	8 (88.89%)	
Menstrual status	Regular	13 (43.33%)	20 (39.22%)	0.896	6 (50.00%)	5 (55.56%)	1
	Irregular	17 (56.67%)	31 (60.78%)		6 (50.00%)	4 (44.44%)	
P53	Wild‐type	27 (90.00%)	44 (86.27%)	0.886	11 (91.67%)	7 (77.78%)	0.787
	Mutant	3 (10.00%)	7 (13.73%)		1 (8.33%)	2 (22.22%)	
Ki‐67	< 20	16 (53.33%)	4 (80.39%)	0.020*	10 (83.33%)	5 (55.56%)	0.364
	≥ 20	14 (46.67%)	10 (19.61%)		2 (16.67%)	4 (44.44%)	
Myometrial invasion	With	19 (63.33%)	7 (13.73%)	< 0.001*	6 (50.00%)	1 (11.11%)	0.160
	Without	11 (36.67%)	44 (86.27%)		6 (50.00%)	8 (88.89%)	
Histological grade	G1	27 (90.00%)	48 (94.12%)	0.807	10 (83.33%)	9 (100.00%)	0.591
	G2	3 (10.00%)	3 (5.88%)		2 (16.67%)	null	

* Represents *p* < 0.05. Numerical data are presented as mean ± standard deviation. Categorical data are presented as numbers (n%).

**TABLE 2:** Univariate and multivariable logistic regression analyses conducted to select clinical features.

Variable	Univariate analysis		Multivariate analysis	
	OR (95% CI)	*p*	OR (95% CI)	*p*
Age	1.229 (0.882, 1.713)	0.307		
BMI	0.968 (0.691, 1.331)	0.871		
HB	1.159 (0.833, 1.611)	0.463		
PLT	0.953 (0.687, 1.323)	0.809		
NEUT	0.817 (0.583, 1.143)	0.323		
LYMPH	1.218 (0.872, 1.701)	0.332		
MONO	0.759 (0.540, 1.067)	0.183		
CA125	0.918 (0.660, 1.276)	0.669		
CA199	1.117 (0.801, 1.559)	0.584		
AFP	1.136 (0.815, 1.584)	0.529		
HE4	0.275 (0.167, 0.451)	< 0.001*	0.431 (0.232, 0.799)	0.025*
ALB	0.903 (0.647, 1.260)	0.614		
ALT	0.906 (0.652, 1.260)	0.623		
AST	1.004 (0.723, 1.392)	0.986		
TB	1.243 (0.886, 1.744)	0.291		
URE	1.260 (0.901, 1.759)	0.256		
CRE	1.216 (0.872, 1.697)	0.333		
Max‐diameter	0.228 (0.135, 0.387)	< 0.001*	0.194 (0.062, 0.608)	0.018*
Parity	0.989 (0.713, 1.373)	0.957		
PCOS	0.408 (0.269, 0.620)	< 0.001*	0.469 (0.286, 0.770)	0.012*
Thyroid disease	0.822 (0.563, 1.200)	0.394		
Diabetes	0.993 (0.715, 1.377)	0.971		
Hypertension	1.057 (0.759, 1.473)	0.784		
Insulin resistance	0.421 (0.292, 0.606)	< 0.001*	0.540 (0.329, 0.886)	0.041*
Menstrual status	1.073 (0.773, 1.489)	0.724		
P53	1.176 (0.842, 1.642)	0.425		
Ki‐67	0.726 (0.519, 1.015)	0.116		
Myometrial invasion	0.332 (0.222, 0.497)	< 0.001*	0.506 (0.299, 0.855)	0.033*
Histological grade	0.767 (0.536, 1.097)	0.223		

Abbreviations: CI, confidence interval; OR, odds ratio.

* Represents *p* < 0.05.

### 3.2 Radiomic Signature Models

**FIGURE 4:**
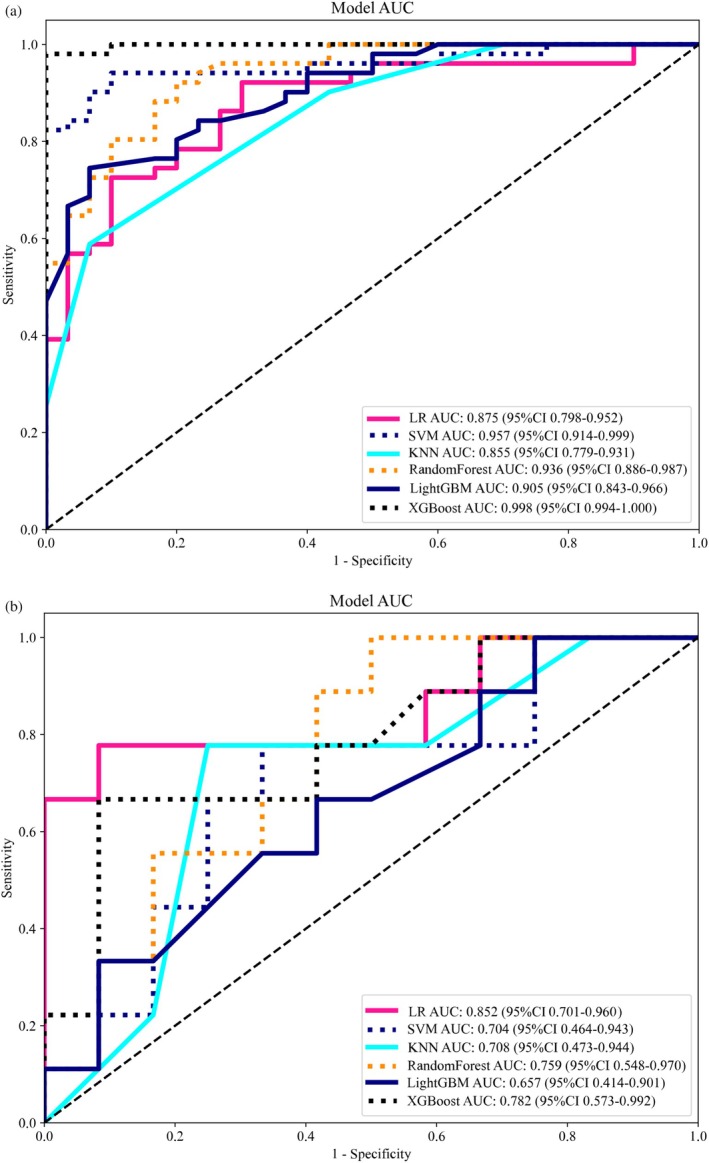
The receiver operating characteristic (ROC) curves of six models in the training (a) and testing (b) cohorts. LR logistic regression, SVM support vector machine, KNN K‐nearest neighbors, RF random forest, XGBoost extreme gradient boosting, LightGBM Light Gradient Boosting Machine.

A total of 1197 radiological features were extracted from MRI images. Following feature scaling and selection, 13 features with nonzero coefficients were retained. Six machine learning methods were employed in this study to construct radiomics models, with the ROC curves for both the training and testing cohorts shown in Figure 4. In the training cohort, all six models achieved AUCs above 0.85, with XGBoost performing best, yielding an AUC of 0.998, an accuracy of 0.975, a sensitivity of 0.961, a specificity of 1.000, a PPV of 1.000, and an NPV of 0.937. However, in the testing cohort, only one radiomics model, the LR model, achieved an AUC above 0.85. The LR model recorded an AUC of 0.852, an accuracy of 0.810, a sensitivity of 0.667, a specificity of 0.917, a PPV of 0.857, and an NPV of 0.786 (Table 3). The results suggest that while the XGBoost model performed well in the training cohort, it showed signs of overfitting. To ensure model stability and generalizability, we ultimately selected the LR model as the optimal radiomics model.

**TABLE 3:** Different models for predicting the effect of fertility preservation in training and test cohorts.

Model		AUC (95% CI)	Accuracy	Sensitivity	Specificity	PPV	NPV
LR	Training	0.875 (0.798, 0.951)	0.878	0.706	0.900	0.923	0.643
	Test	0.852 (0.701, 0.960)	0.810	0.667	0.917	0.857	0.786
SVM	Training	0.957 (0.914, 0.999)	0.914	0.922	0.900	0.940	0.871
	Test	0.704 (0.464, 0.943)	0.667	0.667	0.667	0.600	0.727
KNN	Training	0.855 (0.779, 0.930)	0.531	0.255	1.000	1.000	0.441
	Test	0.708 (0.473, 0.943)	0.571	0.222	0.833	0.500	0.588
RF	Training	0.936 (0.886, 0.986)	0.864	0.902	0.800	0.885	0.828
	Test	0.759 (0.548, 0.970)	0.667	0.889	0.500	0.571	0.857
XGBoost	Training	0.998 (0.993, 1.000)	0.975	0.961	1.000	1.000	0.937
	Test	0.782 (0.572, 0.992)	0.762	0.556	0.917	0.833	0.733
LightGBM	Training	0.905 (0.842, 0.966)	0.802	0.725	0.933	0.949	0.667
	Test	0.657 (0.413, 0.900)	0.571	0.111	0.917	0.500	0.579

Abbreviations: 95% CI, 95% confidence interval; AUC, area under the curve; KNN, K‐nearest neighbors; LightGBM, light gradient boosting machine; LR, logistic regression; NPV, negative predictive value; PPV, positive prediction value; RF, random forest; SVM, support vector machine; XGBoost, extreme gradient boosting.

### 3.3 Combined Model Construction and Validation

**FIGURE 5:**
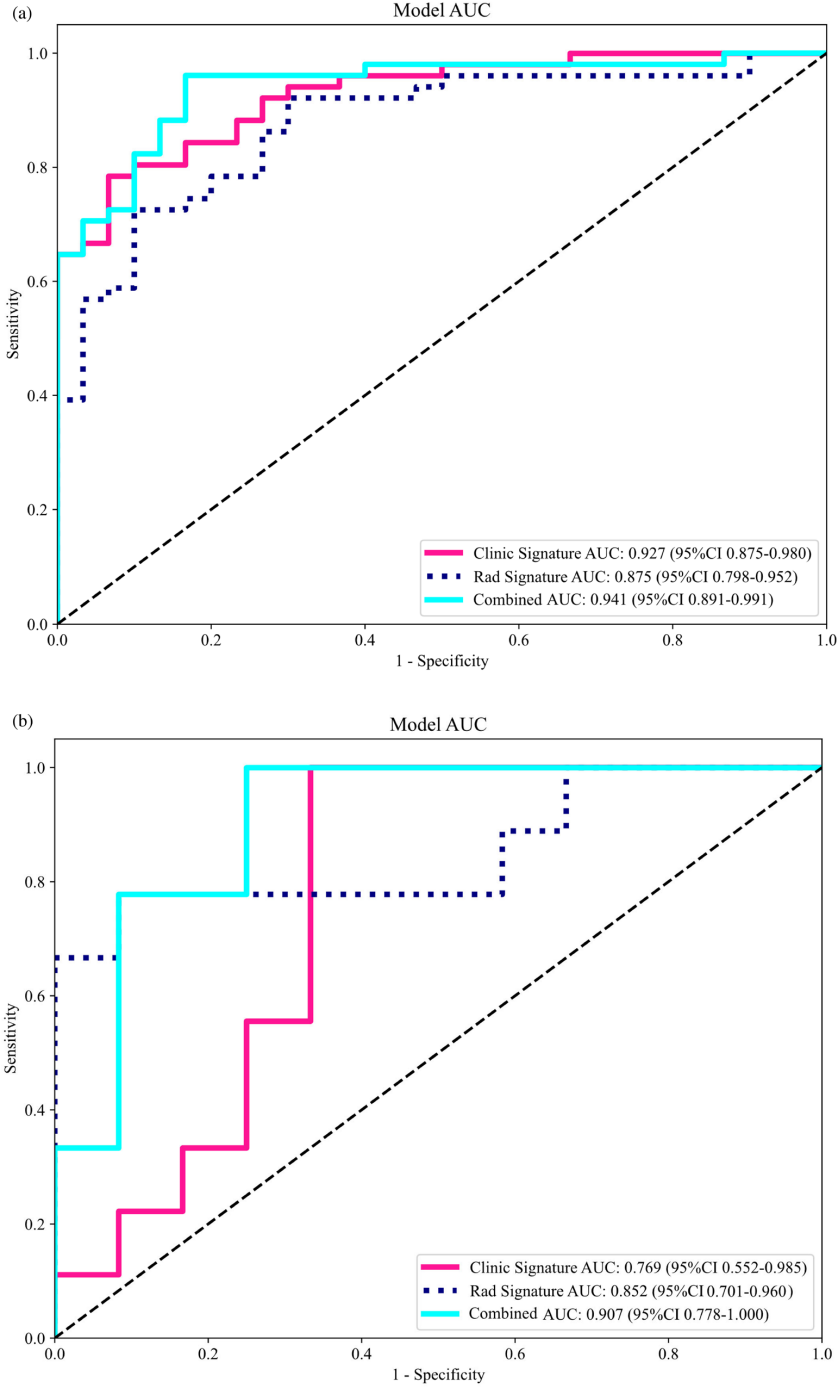
Receiver operating characteristic (ROC) curves for clinical model, radiomics model and combined model in training cohort (a) and testing cohort (b).

**FIGURE 6:**
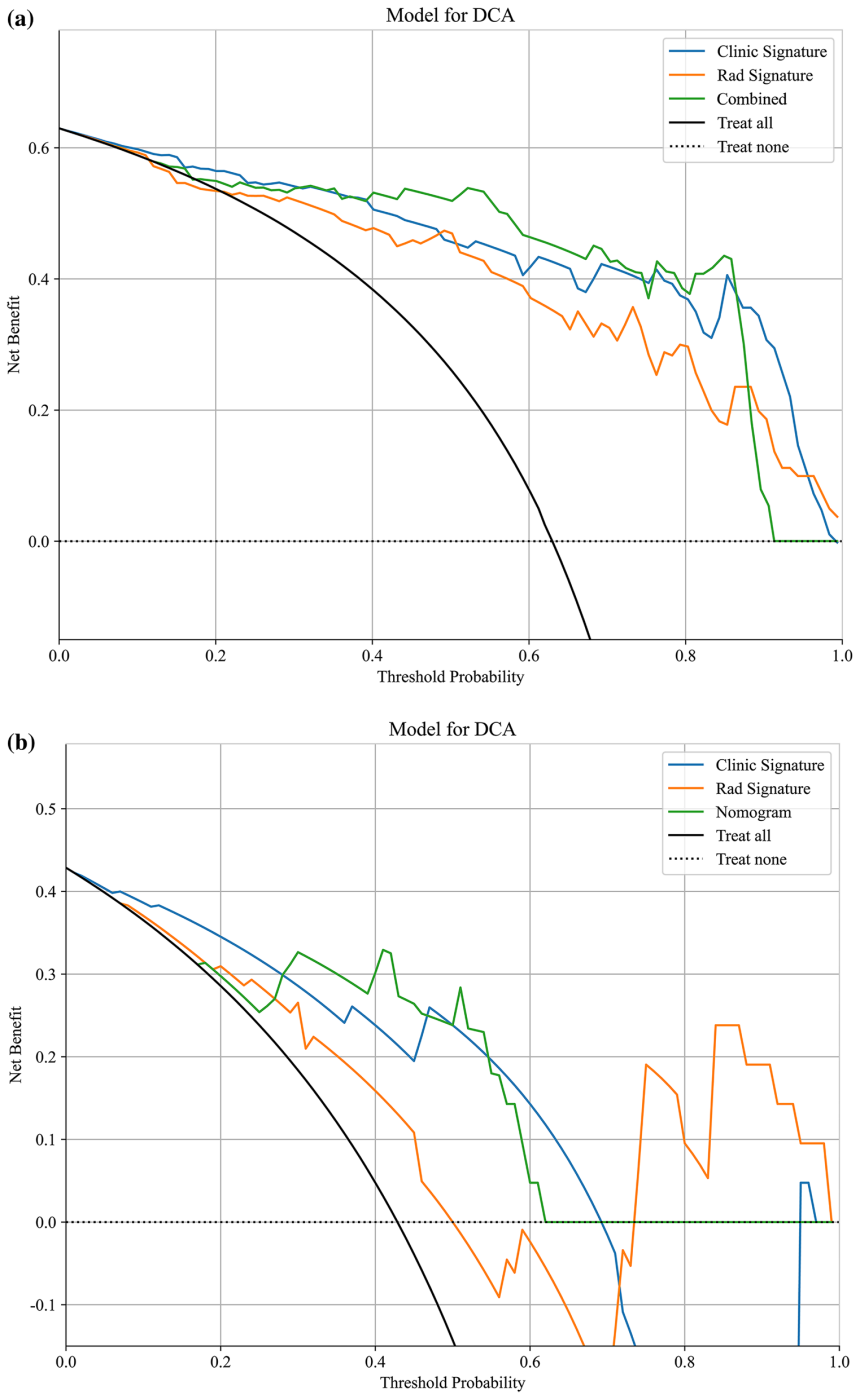
Decision curve analysis (DCA) for three models in training (a) and testing (b) cohorts. Net Benefit = TP/*N* − FP/*N* × pT/(1 − pT), where pT denotes the threshold probability (0.1–0.8). The clinical endpoint was 6 month CR vs. non‐CR. We plotted decision curves comparing the “Fertility‐sparing treatment” and “No fertility‐sparing treatment” strategies.

We developed a combined model by integrating five clinical independent predictors and 13 radiomic features. Clinical, radiomic, and combined models were constructed using logistic regression (LR), and the performance of each model was systematically evaluated. The ROC curves for the three models in both the training and testing cohorts are displayed in Figure 5. In both cohorts, the combined model demonstrated significantly superior discriminative ability compared to the clinical and radiomic models (Table 4), with an AUC of 0.941 in the training cohort and 0.907 in the testing cohort. The DCA curves for the three models are shown in Figure 6. The results suggest that the combined model offered the greatest net benefit in predicting fertility preservation success across both cohorts.

**TABLE 4:** Performance of the clinical model, radiomics model, and combined model in the training and testing cohorts.

Model		AUC (95% CI)	Accuracy	Sensitivity	Specificity	PPV	NPV
Clinical Model*	Training	0.927 (0.874, 0.980)	0.827	0.765	0.933	0.951	0.700
	Test	0.769 (0.552, 0.985)	0.762	0.889	0.667	0.667	0.889
Radiomics Model*	Training	0.875 (0.798, 0.951)	0.878	0.706	0.900	0.923	0.643
	Test	0.852 (0.701, 0.960)	0.810	0.667	0.917	0.857	0.786
Combined Model*	Training	0.941 (0.890, 0.991)	0.901	0.941	0.833	0.906	0.893
	Test	0.907 (0.778, 1.000)	0.810	0.889	0.750	0.727	0.900

Abbreviations: 95% CI, 95% confidence interval; AUC, area under the curve; NPV, negative predictive value; PPV, positive prediction value.

* Represents models that were developed using LR.

## 4 Discussion

### 4.1 Principal Findings

We created a machine‐learning‐based fusion model that integrates clinical predictors and radiomic features using a logistic regression (LR) approach to predict fertility preservation prognosis effectively. Our results demonstrate the model's efficacy, providing clinicians with a feasible and reliable noninvasive tool for assessing the suitability of fertility‐sparing treatments in young patients with endometrial cancer. In recent years, studies examining factors that influence the response rates of fertility‐sparing treatments in endometrial cancer patients have primarily focused on isolated clinical characteristics. To date, no prognostic model aimed at predicting fertility preservation outcomes in endometrial cancer patients has been developed.

### 4.2 Results in the Context of What Is Known

Our findings indicate that among patients who did not achieve remission after 6 months of treatment, the proportion with PCOS and insulin resistance is significantly higher compared to those who achieved remission, corroborating previous research findings [4, 28]. This disparity may be attributed to damage to progesterone receptors in PCOS patients, leading to progesterone resistance [29, 30]. Moreover, the excess endogenous estrogen produced by adipose tissue in insulin‐resistant patients can counteract progesterone therapy [8]. Li et al.'s study further demonstrates that PCOS patients are more susceptible to relapse [12].

Notably, our study identified serum human HE4 level as an independent predictor of fertility‐sparing treatment outcomes. Patients responsive to progestogen therapy exhibited significantly lower baseline serum HE4 levels compared to nonresponders, aligning with Behrouzi et al.'s findings [15]. Recent research has demonstrated a correlation between elevated serum HE4 levels and advanced tumor stage and grade [31]. Furthermore, HE4 has been shown to enhance the proliferation of endometrial cancer cell lines [32, 33]. These findings suggest that HE4 may play a crucial role in promoting tumor invasiveness, proliferation, and resistance to progestogen therapy [34, 35].

### 4.3 Research Implications

Radiomics is an approach that involves extracting and analyzing large quantities of data through the examination of features in medical imaging modalities, including CT and MRI. The fundamental principle of radiomics is to uncover potentially valuable information within imaging characteristics, which can be utilized for disease diagnosis, prognostic assessment, and prediction of treatment outcomes. In our study, the three most important radiomic features are wavelet_LHL_glrlm_ShortRunEmphasis, wavelet_HHL_glcm_Contrast, and log_sigma_30mm_3D_glszm_SmallAreaLowGrayLevelEmphasis. ShortRunEmphasis quantifies the distribution of short run lengths, with higher values indicating shorter run lengths and finer texture. Contrast assesses local intensity variation, with larger values associated with greater intensity differences between adjacent voxels. SmallAreaLowGrayLevelEmphasis evaluates the proportion of smaller areas with lower gray‐level values in the image. These variables suggest that endometrial cancer tumors exhibiting coarser texture, higher gray‐level values, and lower contrast are more prone to developing progestogen tolerance. Similar findings have been observed in studies on myometrial invasion in endometrial cancer [36], suggesting that tumors with poor prognosis are often accompanied by varying degrees of necrosis, hemorrhage, and differences in tissue microstructure between different regions [37] heterogeneities that are typically imperceptible to radiologists through visual inspection alone.

### 4.4 Clinical Implications

Our study demonstrates that the linear LR model outperforms all other models, possibly due to the limited generalization capability and risk of overfitting associated with more complex models when applied to small sample cohorts, coupled with the relatively low number of endometrial cancer patients eligible for fertility preservation [38]. Nevertheless, the LR model achieved impressive performance with fewer training samples while maintaining satisfactory stability and efficiency. Our combined model demonstrated superior predictive performance, followed by the standalone radiomics model, and lastly, the clinical feature model. This finding suggests that radiomic features can provide valuable information on tumor phenotype and microenvironment, complementing other data sources, including clinical characteristics [39]. An integrated model incorporating all features has the potential to yield an accurate and robust evidence‐based clinical decision support system [40].

### 4.5 Strengths and Limitations

This is currently the largest single‐center fertility‐sparing study in endometrial cancer. Furthermore, our study develops the first prognostic model integrating clinical and radiomic features for fertility‐sparing treatment in endometrial cancer, which demonstrates the potential of machine learning in optimizing patient selection for fertility‐preserving approaches and provides a noninvasive tool to assist clinicians in treatment decision‐making for young endometrial cancer patients. This study has several limitations. First, as a retrospective single‐center study, there is a potential for selection bias. Second, manual lesion segmentation is prone to subjectivity and experiential influence [41]; we aim to achieve full automation through deep learning techniques in future studies. Third, we did not extensively extract deep learning‐based features; in future studies, upon collecting a sufficient sample size, we intend to conduct deep learning research in conjunction with traditional radiomics.

## 5 Conclusion

In summary, our research proposes and validates a novel prognostic model for fertility‐preserving treatment in endometrial cancer patients, integrating both clinical and radiomic features derived from MRI. Our study demonstrates that the combined model using LR exhibited superior performance, potentially paving the way for the development of precision medicine approaches and the enhancement of clinical treatment strategies in this field.

## Author Contributions

Wenhan Yuan: conceptualization; data curation; visualization; writing – original draft; writing – review and editing; formal analysis; validation. Haidan Yin: software; visualization. Jianhong Liu: data curation. Ying Zheng: conceptualization; methodology; writing – review and editing; funding acquisition.

## Disclosure

During the preparation of this work, the author used ChatGPT in order to improve the readability and language of the manuscript. After using this tool, the author reviewed and edited the content as needed and takes full responsibility for the content of the publication.

## Ethics Statement

The study was reviewed and approved by the institutional review board of the West China Second Hospital of Sichuan University (IRB No. 2023[024]).

## Consent

Written informed consent was obtained from all the participants prior to the enrollment (or for the publication) of this study.

## Conflicts of Interest

The authors declare no conflicts of interest.

## Data Availability

The data that support the findings of this study are available from the corresponding author upon reasonable request.
